# Comprehensive characterization of B7 family members in breast cancer: B7-H5 switch reverses breast cancer from “immuno-cold” into “immuno-hot” status

**DOI:** 10.1186/s12935-024-03392-4

**Published:** 2024-06-10

**Authors:** Jiayu Liu, Cenzhu Wang, Ying Jiang, Yunxu Zhou, Lingyan Chen, Zhiwen Qian, Lu Liu, Danping Wu, Yan Zhang

**Affiliations:** 1 Department of Oncology, Wuxi Maternal and Child Health Care Hospital, Wuxi School of Medicine, Jiangnan University, No.48 Huaishu Road, Wuxi, Jiangsu 214002 China; 2 Wuxi People’s Hospital, Wuxi Medical Center, The Affiliated Wuxi People’s Hospital of Nanjing Medical University, Nanjing Medical University, Jiangsu, 214000 China; 3 Department of Oncology, The Affiliated Wuxi People’s Hospital of Nanjing Medical University, Jiangsu, 214023 China; 4 Wuxi Maternal and Child Health Hospital, Nanjing Medical University, Jiangsu, 214000 China; 5 Wuxi Maternal and Child Health Hospital, Jiangnan University, Jiangsu, 214002 China

**Keywords:** B7 family, Breast cancer, B7-H5, Tumor immunity, Immuno-hot

## Abstract

The members of the classic B7 family regulate the immune microenvironment of several malignant tumors. However, the potential relationship between the B7 family and the breast cancer (BrCa) tumor immune microenvironment has remained elusive. In the present study, we provide a comprehensive explanation of the expression, clinical significance, mutation, and immune cell infiltration of B7 family molecules in BrCa. First, we recruited 10 patients with BrCa surgery from the Wuxi Maternal and Child Health Hospital and performed single-cell RNA sequencing (scRNA-seq) analysis to investigate the distribution of B7 family members in multiple immune cell subsets. We focused on B7-2, B7-H3, and B7-H5 molecules of the B7 family and constructed tumor microarrays by self-recruiting patients to perform multiple immunohistochemical (mIHC) analyses and study tumor expression of B7-2, B7-H3, B7-H5 and CD8^+^ immune cell infiltration. B7-H5 displayed a strong correlation with CD8^+^ immune cell infiltration. In summary, B7-H5 provides a new perspective for the identification of immunothermal subtypes of BrCa and could function as a switch to reverse BrCa from an “immunologically cold” state to an “immunologically hot” state.

**Graphical abstract:**

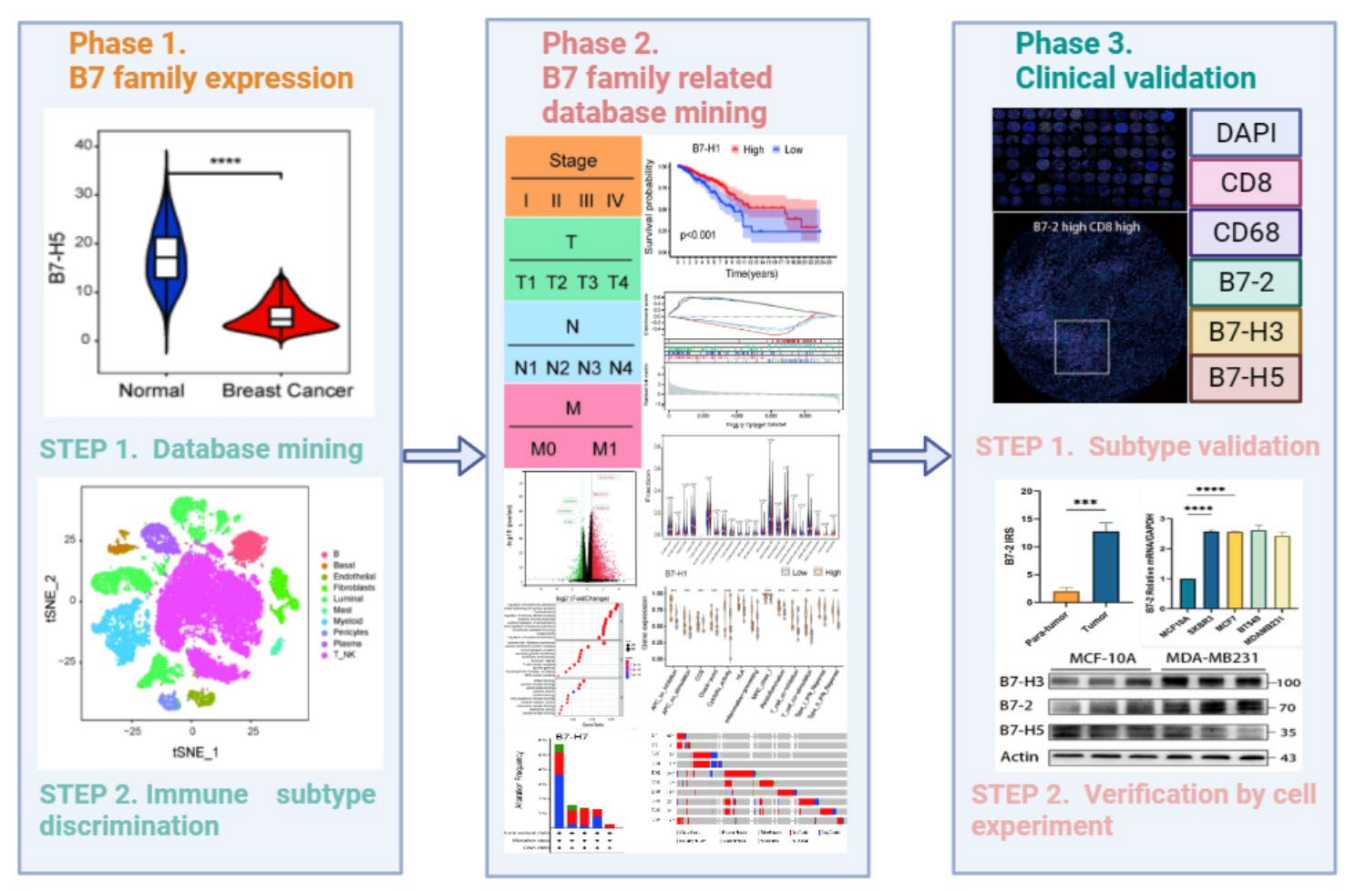

## Introduction

Breast cancer (BrCa) ranks second in mortality among females worldwide, accounting for 31% [1]. Because the traditional treatments of BrCa are limited by several shortcomings, increasing attention has been paid to immunotherapy, which could result in long-term disease control in patients with metastatic BrCa [2]. Immune checkpoint inhibitor (ICI)-based immunotherapy is a cutting-edge method of treating cancer by hindering tumor recurrence [3]. In contrast to chemotherapy and radiation, which indirectly impair cell growth and survival, immunotherapy directly targets the disease by enhancing anticancer immune responses [4]. However, BrCa has not been considered a highly immunogenic malignancy since the 1980s and the overall response rate has remained disappointing when PD-1 or PD-L1-targeted treatments were used alone or in combination with traditional chemotherapy [5]. Consequently, efficient biomarkers are required to categorize patients with BrCa into the best immunotherapy groups and identify those who could benefit from immunotherapy.

Neoadjuvant treatment (NAT) has consistently improved clinical results in patients with BrCa such that a significant percentage of patients who were previously inoperable are now able to undergo surgical resection [6]. A gene expression-based evaluation is crucial for forecasting the course and prognosis of patients with BrCa although it is challenging to precisely estimate a patient’s treatment response to NAT because of the complexity and heterogeneity of BrCa malignancies [7]. Recent research has implicated TIME in controlling BrCa development and NAT effectiveness, and the interplay involves tumor cells, stromal cells, tumor-infiltrating lymphocytes (TILs), and cancer-associated fibroblasts [8]. Tumors’ immunological “immuno-hot and immuno-cold” states are separated according to the nature of TIME. In detail, “immune-hot” tumors consistently exhibit positive therapeutic effects to different treatments, distinguished by effective T cell infiltration and immune support duration. “Immune-cold” tumors are characterized by immunosuppressive duration and resistance to most treatments, including immunotherapy and cytotoxic chemotherapy [9, 10]. Thus, it is crucial to identify “immuno-hot” phenotypes in patients with BrCa because they will benefit more from immunotherapy and have a better prognosis.

The B7 family molecules, which include co-stimulatory and co-inhibitory molecules, intricately regulate immune responses to elicit protective immunity against cancer and infection as well as limit the overactivity of the immune system [11, 12]. B7-1, B7-2, B7-H1, B7-DC, B7-H2, B7-H3, B7-H4, B7-H5, B7-H6, and B7-H7 constitute the B7 family [13]. The common aliases of B7 family members and their respective ligands are shown in Table 1. The immunological responses mediated by T cells can be inhibited or stimulated via the interaction of B7 ligands with different receptors. These substances are involved in tumor evasion and significantly contribute to the activity, differentiation, effective functions, survival, and inhibition of T cell immune response [14, 15]. The signals produced by the B7 family can be manipulated to treat BrCa. However, further research is required to comprehensively understand the possible correlation between B7 family molecules and BrCa, particularly concerning the “immuno-hot” and “immuno-cold” aspects of these molecules and BrCa.

The importance of B7 molecules, particularly B7-H3, B7-2, and B7-H5, in regulating tumor immunity has been widely discussed in recent years. Recent data indicate that B7-H3 is connected to tumor metastasis, proliferation, and treatment resistance, which are associated with a bad prognosis for patients [16]. Furthermore, immunological cells expressing B7-H3 include neutrophils, macrophages, B cells, dendritic cells, myeloid suppressor cells (MDSCs), monocytes, and activated T cells. These findings suggest that B7-H3 regulates TIME [17]. Eltanbouly demonstrated the expression of B7-H5 in human cancer tissues, including those from colorectal, gastric, non-small cell lung, pancreatic, ovarian, prostate, hepatocellular, acute myeloid leukemia, and metastatic melanoma cancers [18]. However, little is known about the expression patterns of B7-H5, B7-H3, and B7-2 in BrCa. In the present study, we systematically characterized the expression patterns and immuno-biological correlations of B7 family members, particularly B7-2, B7-H3, and B7-H5 to investigate immune-related biomarkers and stratify patients into the best immunotherapy strategies. We found a substantial correlation between B7-H5 expression and CD8^+^ T cell infiltration. Consequently, we provide an original strategy to distinguish between the “immuno-hot” and “immune-cold” phenotypes of BrCa. Individuals exhibiting high levels of B7-H5 expression display an immuno-hot phenotype, which may benefit them from immunotherapy.

**Table 1:** Common aliases of B7 family members and their respective ligands

Name	Alternative names	Ligand
B7-1	CD80	CTLA-4, CD28
B7-2	CD86	CTLA-4, CD28
B7-DC	PDCD1LG2, PD-L2, CD273	PD-1
B7-H1	PD-L1, CD274	PD-1
B7-H2	ICOSLG, B7RP1, CD275	ICOS
B7-H3	CD276	-
B7-H4	B7x, B7S1, Vtcn1	-
B7-H5	VISTA, Gi24, VSIR, SISP1	-
B7-H6	NCR3LG1,NR3L1	NKp30
B7-H7	HHLA2	TMIGD2

## Materials and methods

### Single-cell RNA-seq data acquisition and analysis

Single-cell RNA-sequencing (scRNA-seq) was performed by Shanghai Genechem Co., Ltd. on an Illumina HiSeq XTen machine. Cell Ranger 3.0.2 was applied to perform sample demultiplexing, barcode processing, and generate gene count data for each cell. The R package “singleR” package was applied and the Human Primary Cell Atlas Data, Blueprint Encode Data, and Immune Cell Expression Data were used as reference data for auxiliary annotation. This was followed by applying the Cell Marker database and previous studies to identify marker genes for manual annotation of different clusters.

### Acquisition and analysis of TCGA data

Normalized RNA-sequencing and clinical data of BrCa samples were downloaded from the TCGA dataset (https://portal.gdc.cancer.gov/) with clinical features, including Stage, T, N, M and survival outcomes. The association between B7 family and survival outcomes were analyzed via the GEPIA database (http://gepia.cancer-pku.cn/), including overall survival and disease-free survival. The genetic alteration data of B7 molecules was downloaded from the cBioPortal dataset (https://www.cbioportal.org/) including mutation rate, genomic alterations and lollipop plots. TIME analysis was performed using the “e1071”, “parallel”, and “preprocess Core” packages, including TIME scores, immune cell infiltrating and immune functions. The analysis for differential expression of B7 family was conducted via “limma” package while the differentially expressed genes with the criteria of fdr < 0.001 were furtherly analyzed for GO and GSEA functional enrichments through “cluster Profiler,” “org.Hs.eg.db,” “enrichplot,” and “ggplot2” packages. The risk classification of BrCa patients in TCGA database was conducted into two clusters according to B7-H5 and CD8 + immune cell infiltrating via the “Consensus Cluster Plus” package.

### Clinical specimens

The BrCa tissue microarrays (TMA, Cat. T22-0792-149) were obtained from Outdo BioTech (Shanghai, China). The T22-0792-149 TMA contained 63 female BrCa tissues and 20 adjacent tissues, which were used for exploring B7-2, B7-H3, and B7-H5 expression and infiltration of CD8^+^ immune cell infiltration. All patients in the cohort were recruited from the Wuxi Maternal and Child Health Hospital and submitted for mIHC analysis. Detailed clinicopathological characteristics of the cohorts were provided by Freethinking Biotechnology Co., Ltd. (Nanjing, China). Ethical approval for the TMA study was granted by the Clinical Research Ethics Committee, Outdo BioTech (Shanghai, China). The Institutional Review Board of the Wuxi Maternal and Child Health Hospital approved the study’s ethical affairs, and each subject provided written consent (Wuxi Maternal and Child Health Hospital Ethical Review [2021-01-0927-28]).

### MIHC

MIHC staining was performed on the TMAs of BrCa tissues according to the standardized procedure. Ethylenediaminetetraacetic acid (EDTA) was used for antigen retrieval, and the primary antibodies were incubated overnight at 4℃. The primary antibodies used were as follows: anti-B7-2 (1:8000 dilution, Cat. ab219648, clone: EPR20115; Abcam, Cambridge, UK), anti-B7-H5 (1:50 dilution, Cat. ab252438, clone: EPR23665–20, Abcam), anti-CD8 (ready-to-use, Cat. PA067, clone: 457F6F8, Abcarta, Suzhou, China) and anti-B7-H3 (1:200 dilution, Cat. ab134161, clone: EPNCIR122, Abcam).

### Quantitative analysis of mIHC

MIHC staining was used to study the expression of each protein marker after panoramic scanning. The images were analyzed using the halo pathological image analysis software. The signal intensity of the channel was expressed by histochemical score (H-score). H-score ranges from 0 to 300 and is calculated as follows:

$$\eqalign{& H - score\, = \left[ {1 \times *\left( {\% {\rm{ }}cells{\rm{ }}1 + } \right) + {\rm{ }}2 \times *\left( {\% {\rm{ }}cells{\rm{ }}2 + } \right)} \right. \cr & \left. {\quad\quad\quad\quad\quad\quad+ {\rm{ }}3 \times *\left( {\% {\rm{ }}cells{\rm{ }}3 + } \right)} \right] \cr}$$

Each staining channel was used as a unit. The analysis results included statistics on the overall number of positive cells of each channel in each sample and the number of positive cells in the three grades of weak, moderate, and strong, and the corresponding positive cells in the three parts, namely, the nucleus, cytoplasm, and cytomembrane. Finally, we calculated the corresponding percentage in the whole cell and the H-score value of each channel.

### Cell culture

BrCa cell lines SK-BR-3 (Cat. KG197), MDA-MB-231 (Cat. KG033), MCF-7 (Cat. KG031), BT-549 (Cat. KG413), and MCF-10 A (Cat. HH0220) were authenticated using short tandem repeat profiling and obtained from KeyGen (Nanjing, China) and Boster (Wuhan, China). SK-BR-3 cells were cultured in McCoy’s 5 A medium containing 10% fetal bovine serum (FBS) at 37℃ with 5% CO_2_. MCF-7 cells were cultured in Roswell Park Memorial Institute (RPMI)-1640 medium containing 10% FBS at 37 °C with 5% CO_2_, BT-549 cells were cultured in RPMI-1640 medium containing 10% FBS and 0.023 U/mL insulin at 37℃ with 5% CO_2_, and MDA-MB-231 and MCF-10 A cells were cultured in Dulbecco’s Modified Eagle Medium (DMEM) medium containing 10% FBS and 5% HS and 20 ng/mL EGF and 0.5 µg/mL hydrocortisone and 100 ng/mL cholera toxin and 10 µg/mL insulin and 1% P/S at 37℃ with 5% CO_2_. All assays were conducted with mycoplasma-free.

### Real-time quantitative PCR (real-time qPCR)

Real-time quantitative polymerase chain reaction (qPCR) was performed using the Applied Biosystems QuantStudio 5 system (Applied Biosystems; CA, USA). The values were normalized to that of glyceraldehyde 3-phosphate dehydrogenase (GAPDH) and reflected the mean of three separate trials. The 2-ΔΔCt method for quantification was used to assess the data. The primer sequences are shown below (“F” for “forward” and “R” for “reverse”): B7-2 F, 5’-CTGCTCATCTATACACGGTTACC-3’ and R, 5’-GGAAACGTCGTACAGTTCTGTG-3’; B7-H3 F, 5’-TCTGGGCATCCCAAGTTTTGAC-3’ and R, 5’-TCCGCCTTTTGATCTCCGATT-3’; B7-H5 F, 5’ -ACGCCGTATTCCCTGTATGTC-3’ and R, 5’ -TTGTAGAAGGTCACATCGTGC-3’; GAPDH F, 5’ -GGACCTGACCTGCCGTCTAG-3’ and R, 5’ -GTAGCCCAGGATGCCCTTGA-3’.

### Western blotting

The cells were lysed using the radioimmunoprecipitation (RIPA) lysis buffer (Solarbio; Beijing, China), containing phosphatase and protease inhibitors. The protein concentration was measured using the bicinchoninic acid (BCA) protein detection kit (Vazyme; Nanjing, China). Equal amounts of a sample protein (25 µg) were loaded onto a sodium dodecyl sulfate-polyacrylamide gel electrophoresis (SDS-PAGE) gel and then transferred to polyvinylidene fluoride (PVDF) membranes (Beyotime; Shanghai, China). The membranes were blocked for 2 h at normal temperature and incubated overnight at 4℃ with primary antibodies, including anti-B7-2 antibody (Abcam, ab239075, 1:1000), anti-B7-H3 antibody (Abcam, ab134161, 1:1000), anti-B7-H5 antibody (Abcam, ab300042, 1:1000) and anti-β-actin antibody (Abcam, ab8226, 1:1000). Afterward, the membranes were incubated with a suitable secondary antibody (Beyotime) at room temperature for 2 h. Finally, the protein bands were observed using an enhanced chemiluminescence (ECL) reagent (Vazyme).

### Statistical analysis

All statistical analyses were performed using the SPSS V.24.0 (Chicago, Illinois, USA) or GraphPad Prism V.8 (La Jolla, California, USA) software. The correlation between clinical features, immune cell infiltration, and expression of B7-2, B7-H3, and B7-H5 in BrCa samples was studied using either χ^2^ or Fisher’s exact test. Three independent experiments were conducted and the experimental data were analyzed using Student’s *t-*test. For all statistical analyses, *p* ≤ 0.05 was considered significant.

## Results

### Expression and scRNA-seq distribution of B7 family members in BrCa

**Fig. 1:**
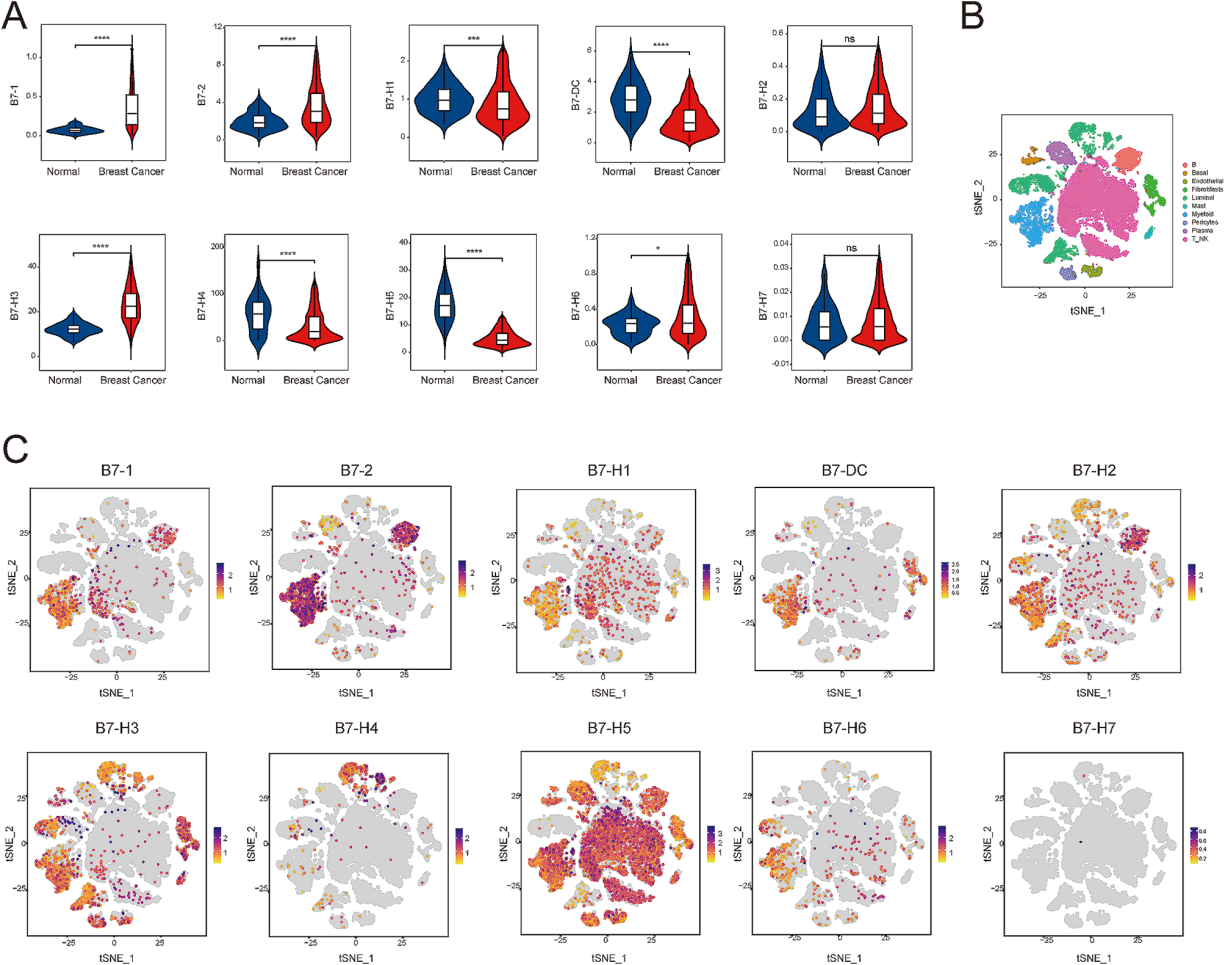
Expression and scRNA-seq distribution of B7 family members in BrCa (**A**) The expression of the B7 family in normal and BrCa tissues using the TCGA database. (**B**) The scRNA-seq analysis of 10 BrCa patients shows the distribution of different cell types in BrCa TIME. (**C**) The relationship between B7 family expression and scRNA-seq distribution in BrCa TIME.

The members of the B7 family and their aliases are shown in Table 1. We initially used the TCGA database to compare the expression of B7 members in normal and BrCa samples. Compared with normal tissues, eight B7 family members demonstrated differential expression in BrCa, including B7-1, B7-2, B7-DC, B7-H1, B7-H3, B7-H4, B7-H5, and B7-H6 (Fig. 1A). In addition, we enrolled 10 BrCa patients from the Wuxi Maternal and Child Health Hospital for scRNA-seq analysis to investigate the distribution of different cell types in BrCa TIME, including B cells, basal cells, endothelial cells, fibroblasts, luminal cells, mast cells, myeloid cells, pericytes, plasma cells, and T/natural killer (NK) cells (Fig. 1B). Next, we explored the association between B7 family member expression and scRNA-seq distribution in BrCa TIME. The cell distribution of B7-H7 was almost invisible. B7-1 and B7-2 were mainly concentrated in myeloid and B cells. The distribution of B7-H1, B7-H2, and B7-H6 was similar, and they were distributed in cell subsets depicted in the diagram. B7-DC was mainly present in myeloid cells and fibroblasts. B7-H4 was mainly found in luminal cells, whereas B7-H3 was significantly enriched in several subgroups except for T/NK and B cells. B7-H5 was largely distributed in all involved cell subsets, except in luminal cells (Fig. 1C).

### Association of B7 family with clinicopathological parameters and survival in BrCa

**Fig. 2:**
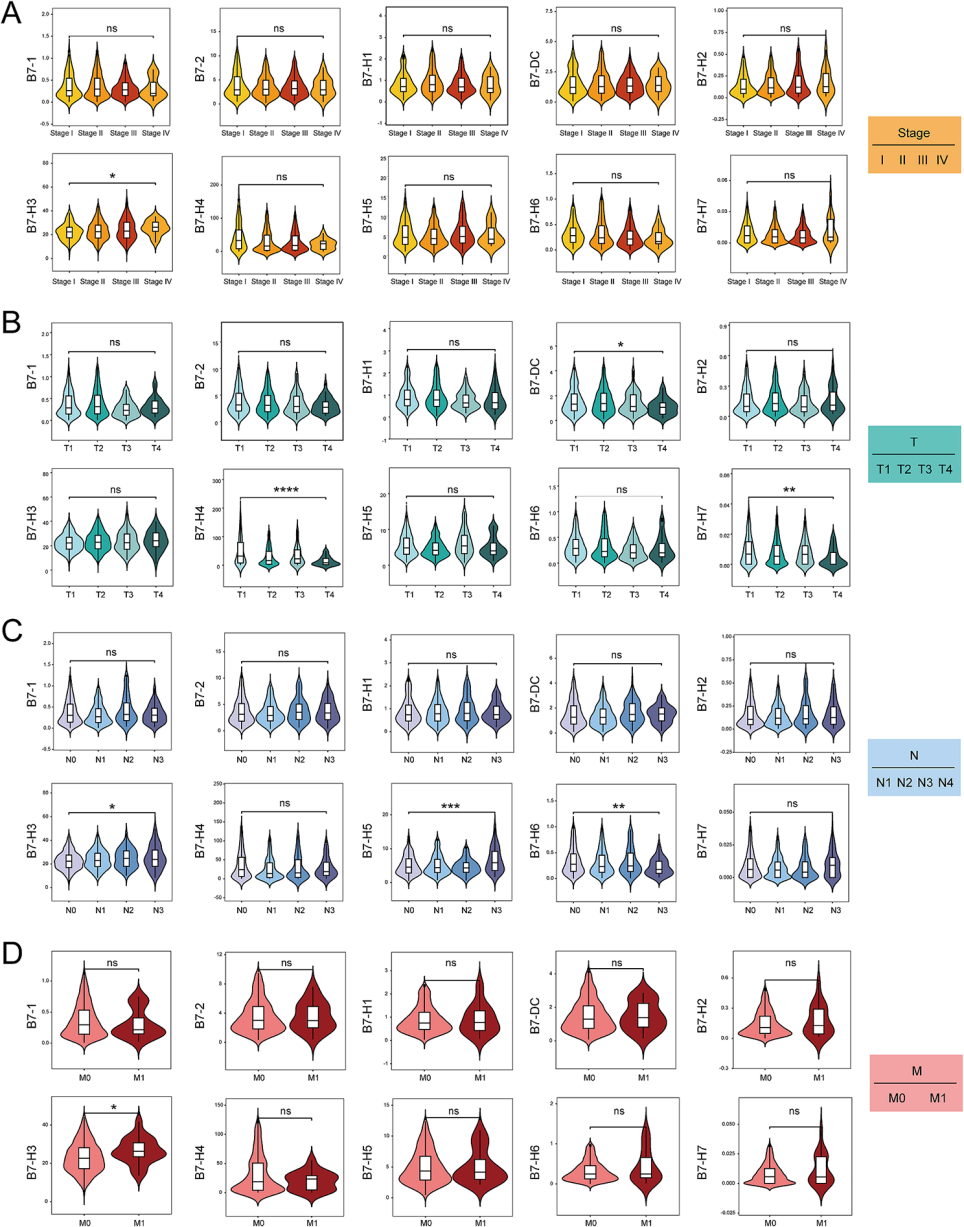
Association between B7 family and clinicopathological parameters and overall survival in BrCa. (**A**) Expression of 10 B7 family members among different TNM stages in BrCa. (**B**) Expression of 10 B7 family members among different T stages in BrCa. (**C**) Expression of 10 B7 family members among different N stages in BrCa. (**D**) Expression of 10 B7 family members among different M stages in BrCa

**Fig. 3:**
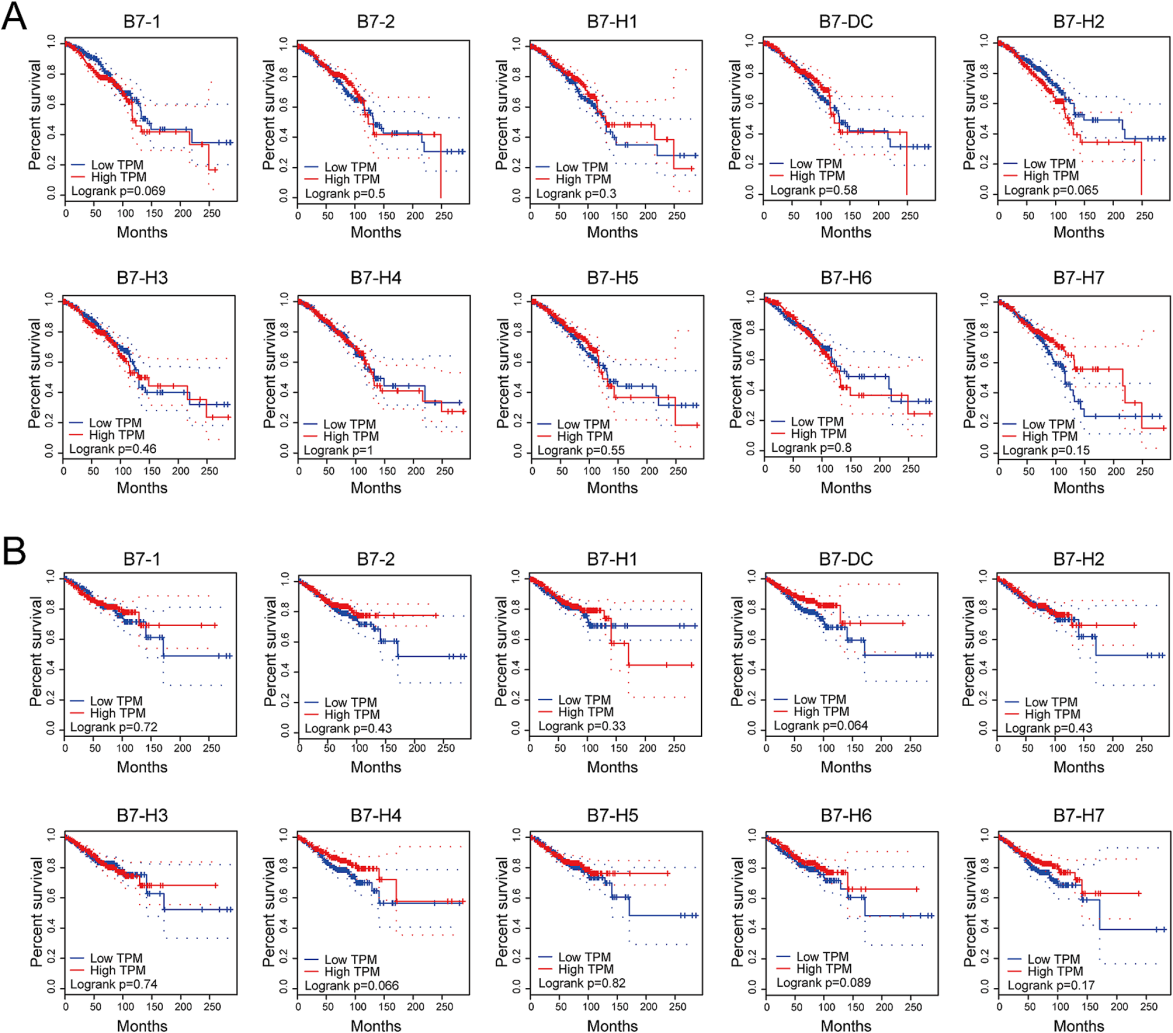
Association between B7 family expression and survival in BrCa. (**A**) Association between B7 family expression and overall survival (OS) in BrCa. (**B**) Association between B7 family expression and disease-free survival (DFS) in BrCa

We next analyzed the correlation between B7 family members and clinicopathological parameters in BrCa, including TNM stage, T stage, N stage, and M stage (Fig. 2). According to the TNM stage, B7-H3 demonstrated significant differential expression among different TNM stages (*p* < 0.05). In addition, based on the T stage, B7-DC (*p*<0.05), B7-H4 (*p*<0.0001), and B7-H7 (*p*<0.01) demonstrated significant differential expression among different T stages. In addition, the expression of B7-H3 (*p* < 0.05), B7-H5 (*p* < 0.001), and B7-H6 (*p* < 0.01) was related to the N stage, whereas the expression of B7-H3 (*p* < 0.05) correlated with the M stage. Further, we investigated the relationship between the B7 family and survival in BrCa, including overall survival (OS) and disease-free survival (DFS). Thus, no significant association was present between B7 family members and patients with OS and DFS (See Fig. 3).

### Functional enrichment and signaling pathways of B7 family members in BrCa

**Fig. 4:**
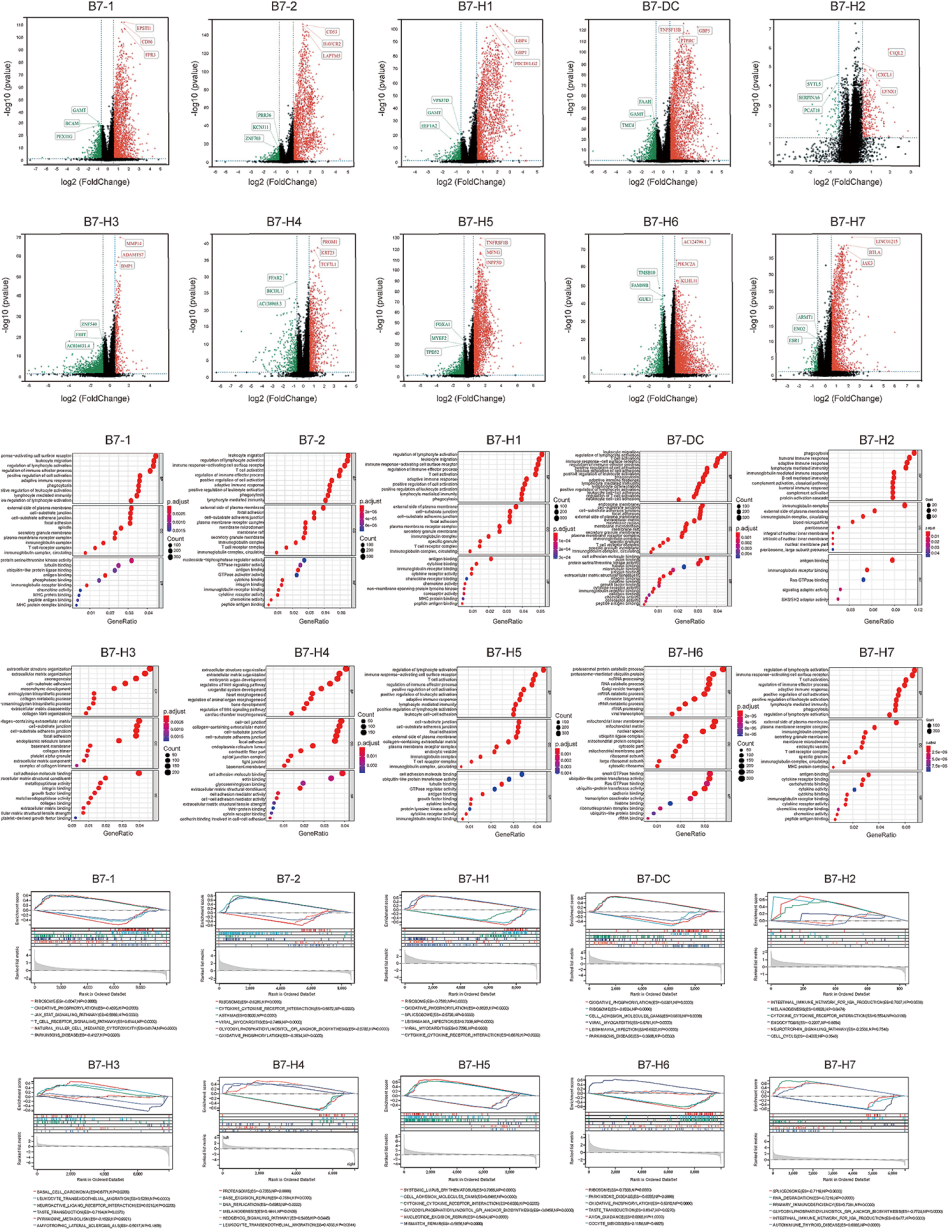
Functional enrichment and signaling pathways of B7 family members in BrCa. (**A**) The volcano plot depicts differentially expressed genes of B7 family members in BrCa. (**B**) The GO functional enrichment analysis of B7 family members in BrCa. (**C**) The GSEA signaling pathway analysis of B7 family members in BrCa

The median of B7 family members was used to perform differential analysis between low-expressed and high-expressed groups, whereas differentially expressed genes of B7 family members were presented using the volcano plot (Fig. 4A). Next, we conducted the gene ontology (GO) enrichment analysis of B7 family members, including biological process (BP), cellular component (CC), and molecular function (MF) (Fig. 4B). The gene set enrichment analysis (GSEA) enrichment analysis of B7 family members was performed for potential signaling pathways (Fig. 4C).

B7-1 was enriched in immune response-activating cell surface receptors, external side of the plasma membrane, and protein serine or threonine kinase activity, whereas B7-1 was enriched in ribosomes, oxidative phosphorylation, JAK–STAT signaling pathway, T cell receptor signaling pathway, NK cell-mediated cytotoxicity, and Parkinson’s disease. Similarly, B7-2 was enriched in leukocyte migration, the external side of the plasma membrane, and nucleoside-triphosphatase regulator activity, whereas B7-2 was enriched in ribosomes, cytokine, asthma, viral myocarditis, glycosylphosphatidylinositol (GPI) anchor biosynthesis, and oxidative phosphorylation. B7-H1 was enriched in the regulation of lymphocyte activation, the external side of the plasma membrane, and antigen binding, whereas B7-H1 was enriched in the ribosomes, oxidative spliceosomes, *Leishmania* infection, viral myocarditis, and cytokine–cytokine receptor interaction. B7-DC was enriched in leukocyte migration, endosome membrane, and cell adhesion molecule (CAM) binding, whereas B7-DC was enriched in oxidative phosphorylation, ribosome, CAMs, viral myocarditis, *Leishmania* infection, and Parkinson’s disease. B7-H2 was enriched in phagocytosis, immunoglobulin complex, and antigen binding, whereas B7-H2 was enriched in intestinal immune network for IGA production, melanogenesis, cytokine, cytokine receptor interaction, endocytosis, neurotrophin signaling pathway, and cell cycle. B7-H3 was enriched in extracellular structure organization, collagen-containing extracellular matrix, and CAM binding, whereas B7-H3 was enriched in basal cell carcinoma, leukocyte, neuroactive ligand–receptor interaction, taste transduction, pyrimidine metabolism, and amyotrophic lateral sclerosis. B7-H4 was enriched in extracellular structure organization, cell–cell junction, and CAM binding, whereas B7-H4 was enriched in the proteasome, base excision repair, DNA replication, melanogenesis, hedgehog signaling pathway, and leukocyte transendothelial migration. B7-H5 was enriched in the regulation of lymphocyte activation, cell–substrate junction, and CAM binding, whereas B7-H5 was enriched in systemic lupus erythematosus, CAMs, cytokine–cytokine receptor interaction, GPI anchor biosynthesis, nucleotide excision repair, and mismatch repair. B7-H6 was enriched in proteasomal protein catabolic process, mitochondrial inner membrane, and small GTPase binding, whereas B7-H6 was enriched in ribosomes, Parkinson’s disease, oxidative phosphorylation, taste transduction, axon guidance, and oocyte meiosis. B7-H7 was enriched in the regulation of lymphocyte activation, the external side of the plasma membrane, and antigen binding, whereas B7-H7 was enriched in spliceosomes, RNA degradation, primary immunodeficiency, GPI anchor biosynthesis, intestinal immune network for IGA production, and autoimmune thyroid disease.

### Genomic alterations of B7 family members in BrCa

**Fig. 5:**
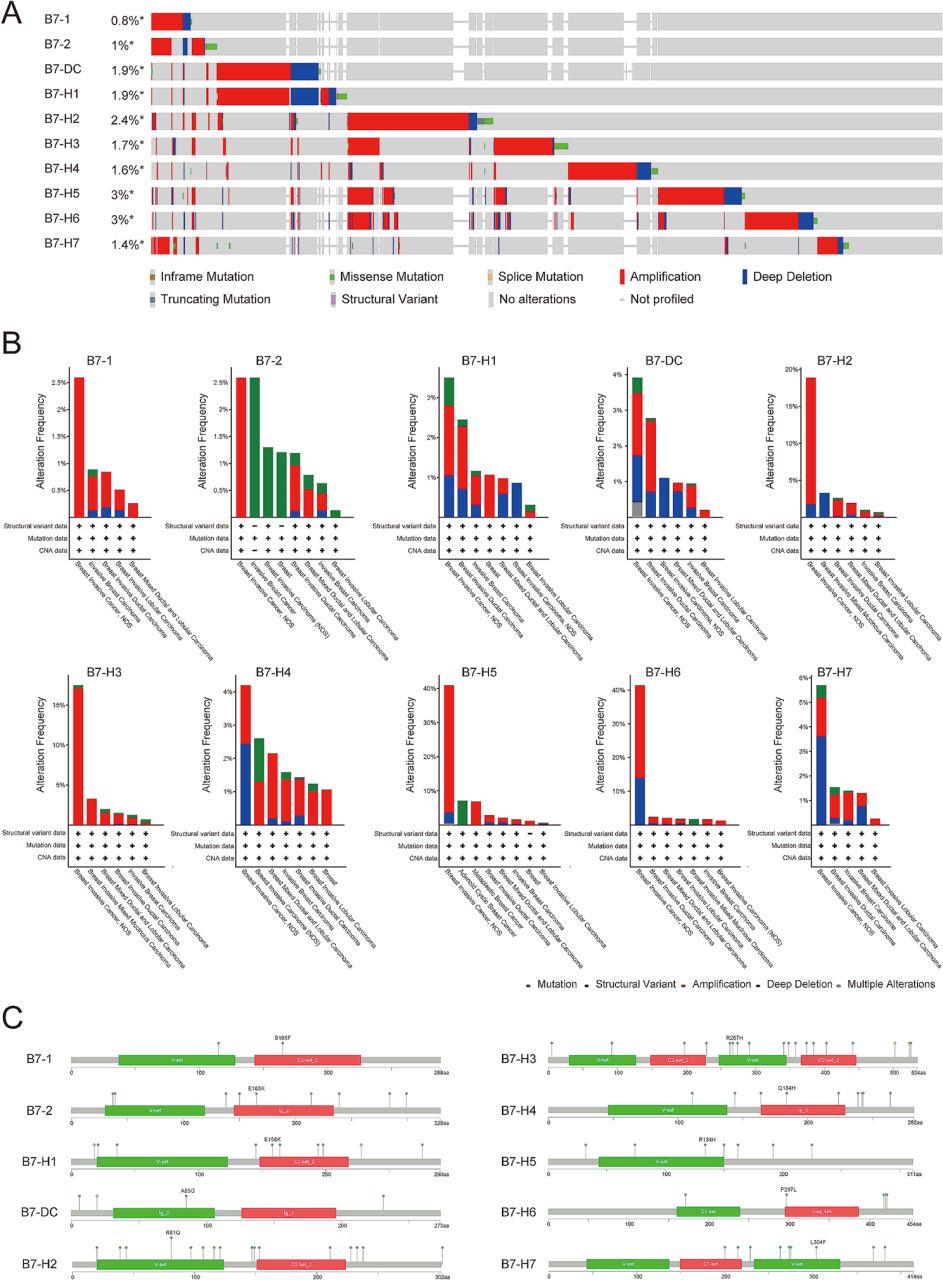
Genomic alterations of B7 family members in BrCa. (**A**) The mutation rate of B7 family members in BrCa. (**B**) The genomic alterations of B7 family members in different BrCa-related researches. (**C**) The lollipop plot of B7 family members in BrCa

We used the cBioPortal database to investigate the relationship between B7 family members and genomic alterations in BrCa. With respect to the genomic alteration frequency, the mutation ratios of B7-H5 and B7-H6 were relatively higher (up to 3%), whereas the mutation rates of B7-1 and B7-2 were lower, less than 1% (Fig. 5A). Genomic alterations of B7 family members in different BrCa-related researches were presented, including structural variant data, mutation data, and CNA data (Fig. 5B). In addition, the lollipop plot of B7 family members in BrCa is depicted (Fig. 5C).

### Correlation between B7 family members and TIME in BrCa

**Fig. 6:**
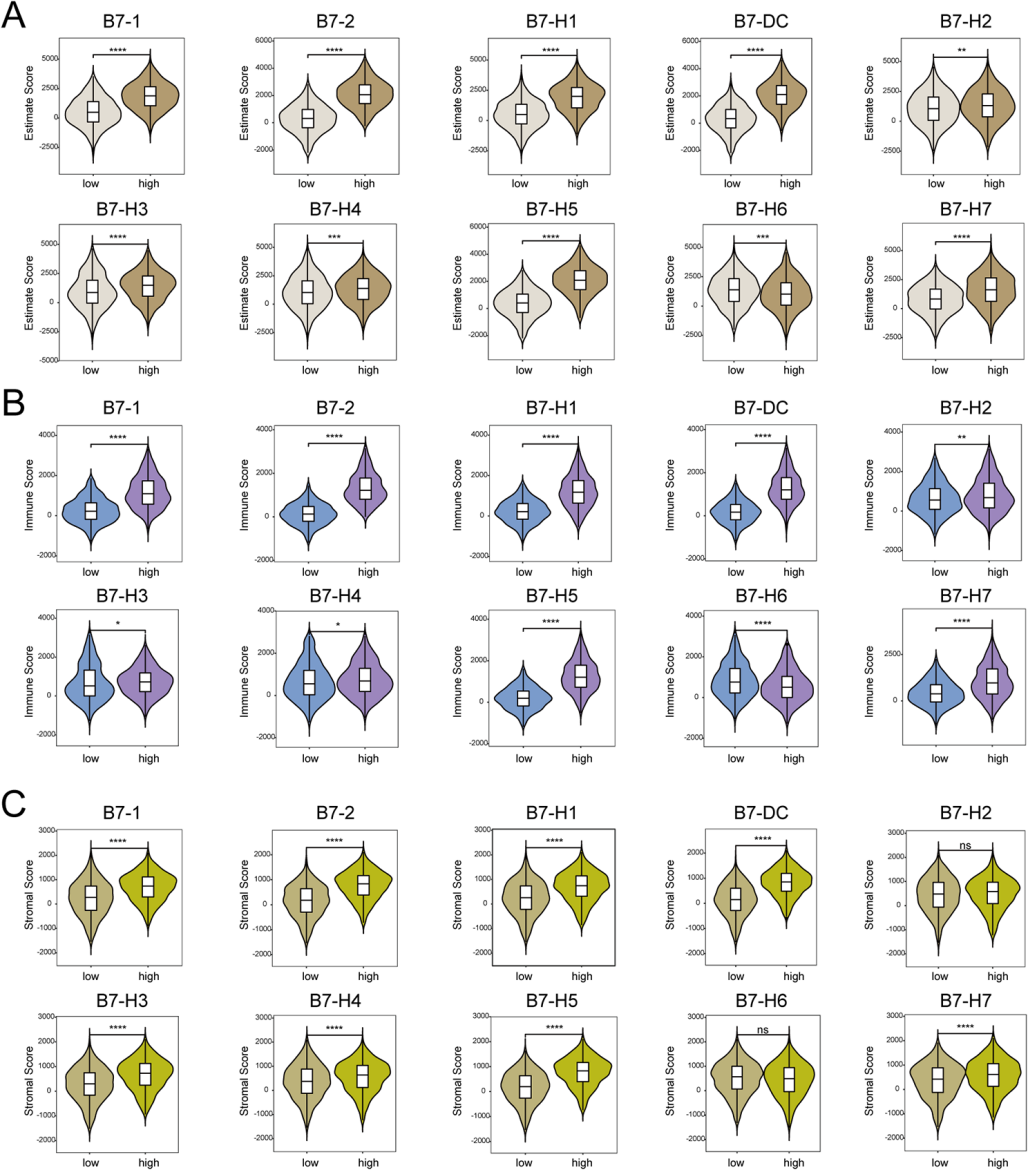
Correlation between B7 family members and TIME in BrCa. Relationship between B7 family members and TIME estimate score. (**B**) Relationship between B7 family members and TIME immune score. (**C**) Relationship between B7 family members and TIME stromal score

**Fig. 7:**
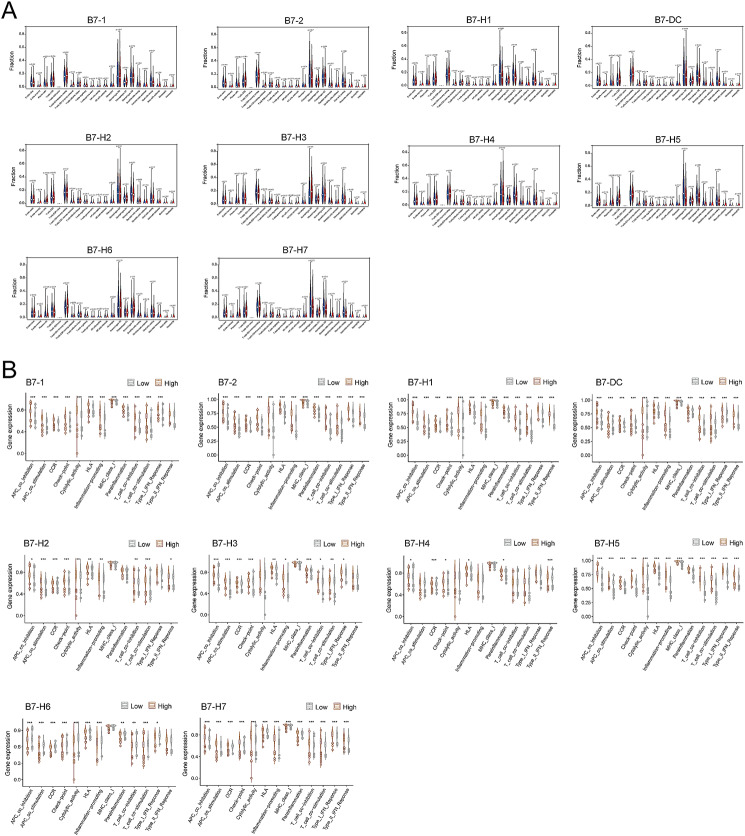
Correlation between B7 family members and immune cell infiltration and immune functions. (**A**) Relationship between B7 family members and immune cell infiltration. (**B**) Relationship between B7 family members and immune function

We next analyzed the relationship between the B7 family and TIME in BrCa, including TIME scores, immune cell infiltration, and immune functions. TIME score included estimate score, immune score, and stromal score, whereas the immune cell infiltration included 23 kinds of immune cells. The immune functions consisted of antigen-presenting cell (APC) co-inhibition, APC co-stimulation, CCR, checkpoint, cytolytic activity, HLA, promotion of inflammation, MHC class I, parainflammation, T cell co-stimulation, T cell co-inhibition, type I interferon (IFN) response, and type II IFN response. According to the TIME scores, 10 B7 family members exhibited significant differences in estimate scores and immune scores, whereas 8 B7 family members displayed significant differences in stromal scores, except for B7-H2 and B7-H6 (Fig. 6A-C). In addition, the relationship between B7 family members and immune cell infiltration, and immune functions were presented (Fig. 7A-B).

### Expression of B7-2, B7-H3, and B7-H5 in BrCa

**Fig. 8:**
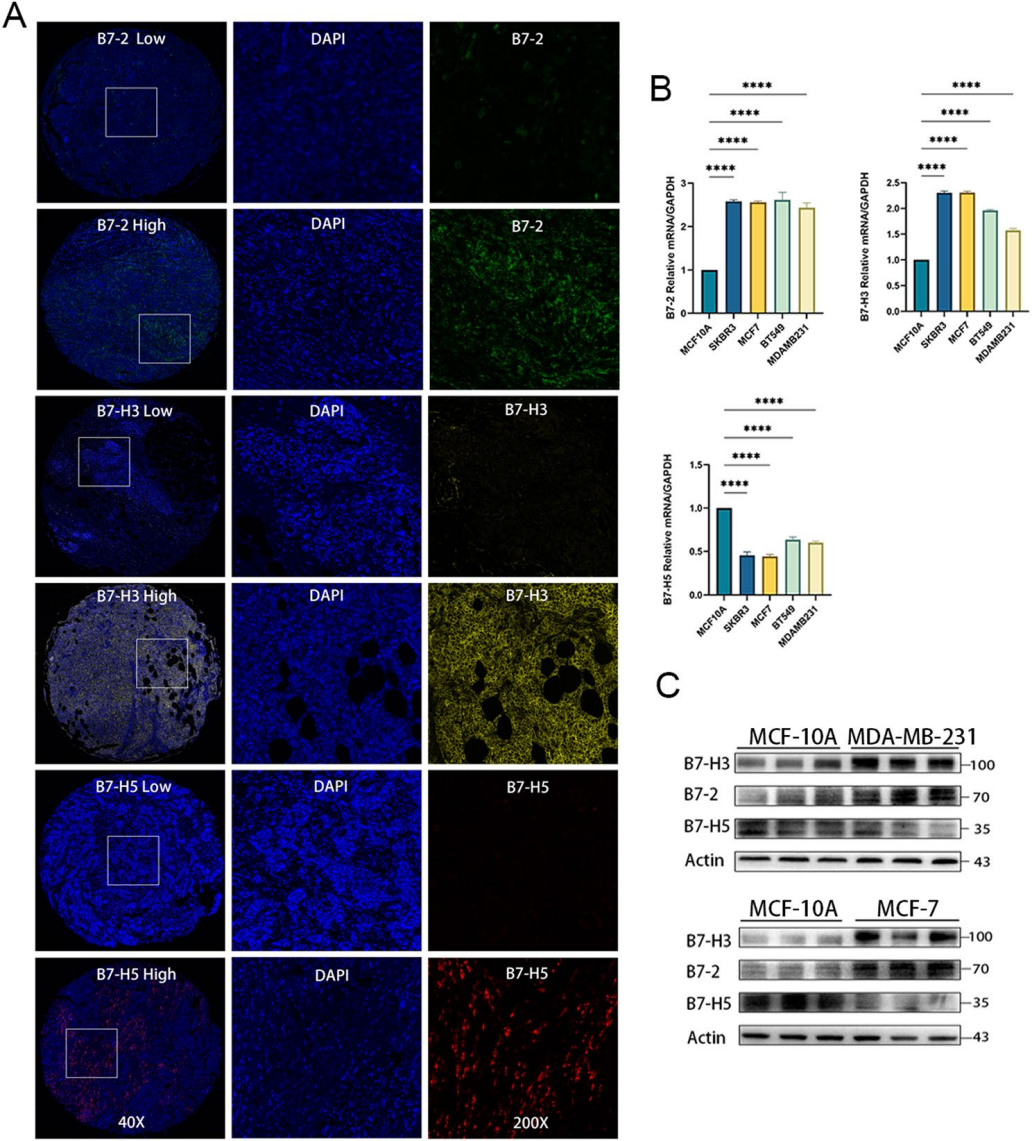
B7-2, B7-H3, and B7-H5 expression in BrCa. (**A**) Representative images of B7-2, B7-H3, and B7-H5 expression in BrCa tissues stained by mIHC. All representative images were divided into low expression group and high expression group. The DAPI staining results are listed as a reference. (**B**) The expression of B7-2, B7-H3, and B7-H5 in several BrCa cells was assessed by qRT-PCR. (**C**) The expression of B7-2, B7-H3, and B7-H5 in several BrCa cells was assessed by western blotting. Normal breast cell line MCF-10 A was used as a reference. Significance was calculated using Student’s *t*-test

We recruited a clinical cohort from the Wuxi Maternal and Child Health Hospital. The preliminary analysis revealed the correlation between the B7 family and the clinical stage. B7-H3 and B7-H5, two molecules with clinical research potential, as well as B7-2, a B7 family member with little research in BrCa, were selected for immunohistochemical and mIHC analyses. Figure 8A depicts representative images of B7-2, B7-H3, and B7-H5 expression in mIHC-stained tumor tissue. Representative images stained with B7-2, B7-H3, and B7-H5 were divided into low expression group and high expression group for display. DAPI staining results were used as a reference. Statistical analysis of H-score staining results in BrCa microarray tumor (*n* = 63) and adjacent tumor (*n* = 20) tissues demonstrated that the expression of B7-2 and B7-H3 in BrCa tissues was significantly higher than in adjacent tumor tissues, whereas no statistical difference was found in B7-H5 between adjacent tumor tissues and BrCa tissues (Fig. 8B). In addition, we used multiple subtypes of BrCa cells to further verify, whereas we used normal breast cells MCF-10 A as controls. The results of western blotting and qRT-PCR were consistent, indicating that B7-2 and B7-H3 were highly expressed in BrCa, and the expression of B7-H5 was low in BrCa (Fig. 8C-D).

According to the clinical cohort in Wuxi Maternal and Child Health Hospital, the relationship between clinical features and expression of B7-2, B7-H3, and B7-H5 was analyzed using the chi-square test or Fisher’s test. The statistical results demonstrated that B7-2, B7-H3, and B7-H5 in BrCa tissues were not statistically correlated with clinical features (Table 2).

**Table 2:** Association between B7-2, B7-H3, B7-H5, and clinical features, infiltration of CD8+immune cells

**Features**		No.	B7-2		*X* ^*2*^	*P* value	**B7-H3**		*X* ^*2*^	*P* value	**B7-H5**		*X* ^*2*^	*P* value
			Low	High			Low	High			Low	High		
**Age(years)**					1.985	0.159			0.013	0.91			1.985	0.159
	≤ 50	35	15	20			18	17			15	20		
	> 50	28	17	11			14	14			17	11		
**T**					1.007	1			3.232	0.164			1.007	1
	T1	29	15	14			12	17			15	14		
	T2	33	17	16			20	13			17	16		
	T3	1	0	1			0	1			0	1		
**N**					1.942	0.615			2.138	0.563			1.209	0.81
	N0	27	15	12			14	13			14	13		
	N1	24	13	11			14	10			13	11		
	N2	8	3	5			3	5			4	4		
	N3	4	1	3			1	3			1	3		
**TNM Stage**					1.829	0.401			8.586	0.014*			0.829	0.661
	Stage I	15	8	7			4	11			7	8		
	Stage II	36	20	16			24	12			20	16		
	Stage III	12	4	8			4	8			5	7		
**Subtype**					6.108	0.094			3.35	0.346			2.426	0.507
	Luminal A	4	3	1			2	2			3	1		
	Luminal B	26	10	16			16	10			11	15		
	Her2-enriched	20	14	6			10	10			12	8		
	TNBC	13	5	8			4	9			6	7		
**CD8+**					3.566	0.059			1.916	0.166			19.437	< 0.001***
	low	32	20	12			19	13			25	7		
	High	31	12	19			13	18			7	24		
	High	25	5	20			11	14			8	17		

### B7-H5 is significantly correlated with CD8^+^cell infiltration and has the potential to predict immunothermal tumors

**Fig. 9:**
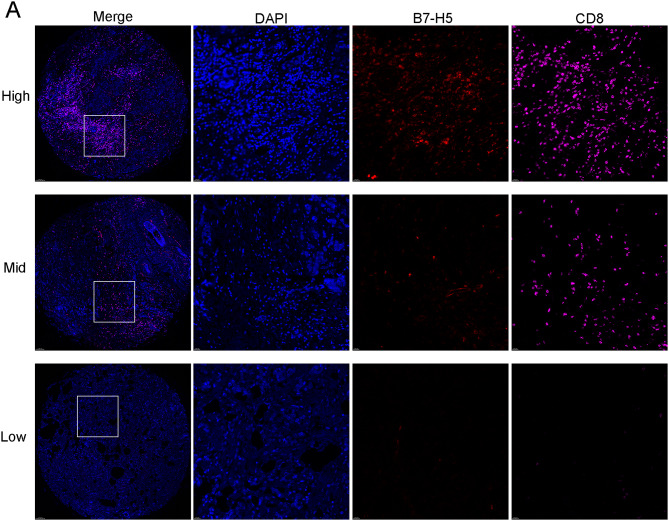
B7-H5 and CD8 + expression in BrCa. (**A**) Representative images uncovering B7-H5 and CD8^+^ expression in tumor tissues using mIHC staining. The images are divided into three levels: B7-H5 low and CD8^+^ low, B7-H5 mid and CD8^+^ mid, and B7-H5 high and CD8^+^ high. The DAPI staining results are used as a reference

CD8^+^ immune cells were divided into three layers with high, medium and low expression. We used the clinical cohort in Wuxi Maternal and Child Health Hospital to study the relationship between infiltration of CD8^+^ immune cells and expression of B7-2, B7-H3, and B7-H5 using chi-square test or Fisher’s test. We found that B7-H5 significantly correlated with CD8^+^ cells (*p* < 0.001), as shown in Table 2. We demonstrated the representative images of B7-H5 and CD8 high, middle, and low layers. As depicted in Fig. 9A, the chip diagram (40 ×) uses a white box to select typical mIHC images, and four single-channel display images of DAPI\B7-H5\CD8+ (200 ×) are conducted. Results demonstrated that CD8^+^ infiltration increased significantly with an increase in B7-H5 expression. We believe that B7-H5 is a potential molecule in predicting thermal tumors, that is, immune invasive tumors.

**Fig. 10:**
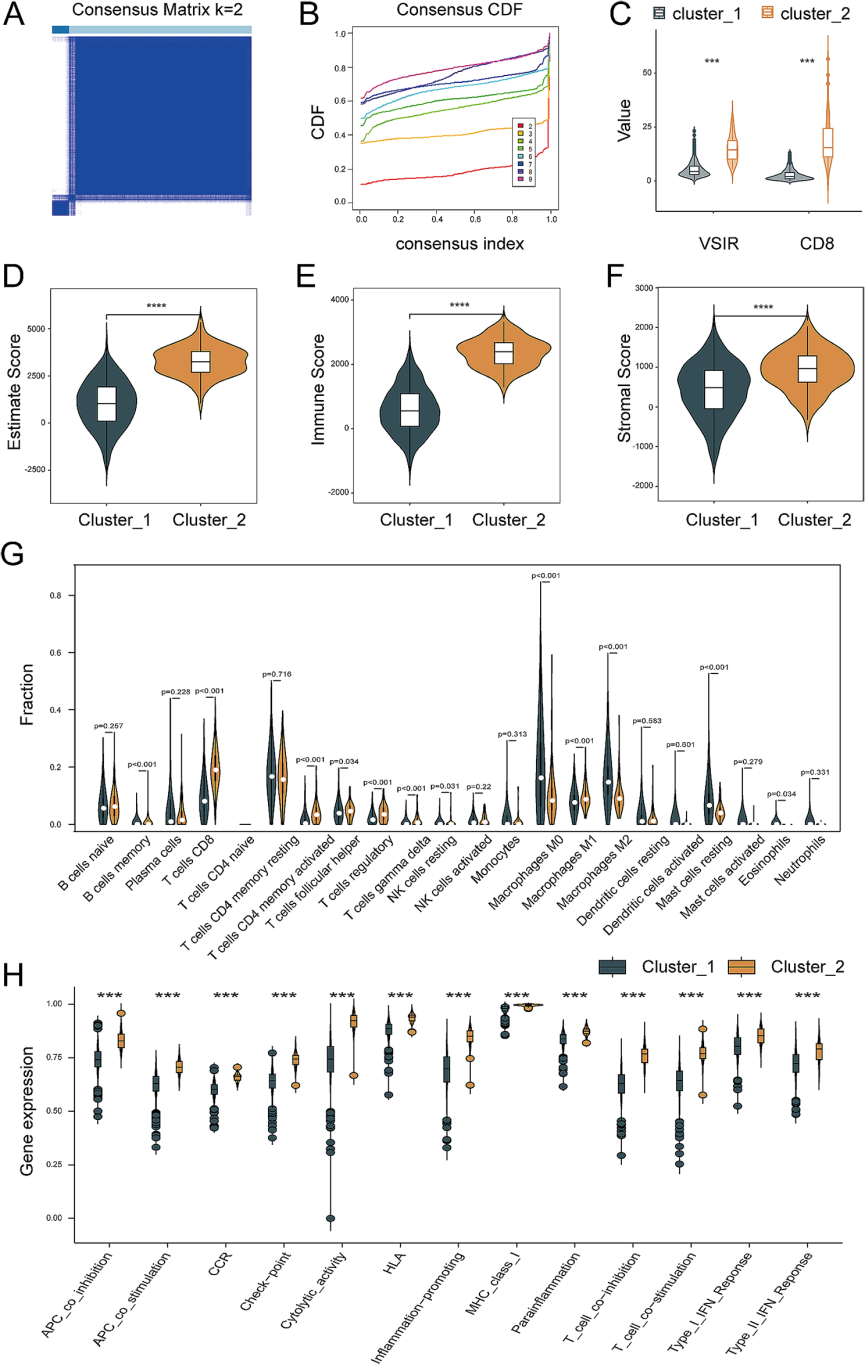
Association between B7-H5 expression and CD8^+^ immune cell infiltration in BrCa patients. (**A**-**C**) Risk classification of patients with BrCa according to B7-H5 and CD8^+^ expression. (**D**) TIME estimate score of risk classification in BrCa. (**E**) TIME immune score of risk classification in BrCa. (**F**) TIME stromal score of risk classification in BrCa. (**G**) Immune cell infiltration of risk classification in BrCa. (**H**) Immune functions of risk classification in BrCa

We performed a cluster analysis to divide the expression of B7H5 and CD8 into cluster 1 and cluster 2, in which cluster 2 referred to high expression of B7H5 and high CD8^+^ (Fig. 10A-C). The risk classification of patients with BrCa was performed based on the expression of B7-H5 and CD8^+^, which was further assessed in terms of TIME, degree of immune cell infiltration, and immune functions (Fig. 10D-F).

## Discussion

The B7 family has recently received considerable attention. Immunomodulatory B7 family proteins are significantly expressed in different malignancies and are known to regulate a disease’s advancement and unfavorable prognosis [12, 19]. The expression of B7 family members could be a useful predictive indicator for women with BrCa who are diagnosed and treated early. Increasing evidence suggests that B7 molecular expression dysregulation inhibits TIME and immune escape. In this work, we first studied the expression patterns of these B7 molecules in BrCa. Compared with normal tissues, eight B7 family members displayed differential expression in BrCa, including B7-1, B7-2, B7-DC, B7-H1, B7-H3, B7-H4, B7-H5, and B7-H6. Furthermore, the B7 family members are genetically stable and change with a low frequency in BrCa.

B7-H3 is a transmembrane immunoregulatory protein that is physically linked to B7 family proteins. It is a negative immunomodulatory protein, which is upregulated in different human malignant tumors and downregulated in normal tissues [20]. It is reported that B7-H3 knockdown increases the chemosensitivity of mantle cell lymphoma [21]. In addition, B7-H3 silencing increases drug-induced cytotoxicity and drug-induced apoptosis in acute monocytic leukemia (AML) cells via upregulating the caspase-3 (CASP-3) activity in vitro [22]. Artemether derivatives derived from Chinese herbs can treat a range of malignant tumors. Research has demonstrated that doxorubicin could suppress B7-H3 expression in neuroblastoma cell lines, whereas B7-H3 suppression further inhibited tumor development in cells treated with artemether [23]. The B7-H3-mediated treatment resistance in melanoma is associated with the activation of the P38–MAPK pathway [24]. Chemoresistance in ovarian cancer is attributed to B7-H3 expression, which stimulates the PI3K/AKT signaling pathway and upregulates Bcl-2 in protein levels [25, 26]. Thus, B7-H3 has emerged as a viable therapeutic target for cancer.

B7-2 functions as a ligand for CD28 and cytotoxic T lymphocyte antigen 4 (CTLA-4) [27]. B7-2 is expressed in dendritic cells, B/T cells, and APCs, whereas APCs have been shown to upregulate B7-2 expression under stimulatory circumstances [28]. CD28 has a high affinity for B7-2, whereas CTLA-4 has an even higher affinity for both B7-1 and B7-2 [29, 30]. A few studies have been conducted on the prognostic and clinical importance of B7-2 in malignant tumors. Soluble B7-2 (sB7-2) is present in the serum of healthy individuals and is present in resting monocytes, dendritic cells, and certain cancer cells [31, 32]. Elevated sB7-2 levels have been reported in different leukemias, including acute myeloid leukemia (AML), and B-chronic lymphocytic leukemia (B-CLL) [32–34]. Patients with myelodysplastic syndrome (MDS) exhibit greater levels of sB7-2. Compared to AML patients with normal B7-2 levels, those with higher levels of B7-2 had lower chances of complete response and shorter survival [35]. Furthermore, the anti-tumor response demonstrated by B7.2-IgG was CD8^+^ dependent.

The inclusion of B7-H5, a T-cell-activated V-domain immunoglobulin inhibitor, could be a viable target for immune oncology [36, 37]. B7-H5 is mostly expressed in hematopoietic tissue and tissues with a high concentration of white blood cells. In comparison to its decreased expression on CD4^+^ and CD8^+^ T lymphocytes, B7-H5 is overexpressed in bone marrow cells. B7-H5 expression is absent in B cells. However, it was highly expressed in plasma cells [18, 38, 39]. Because of its main homeostatic function, B7-H5 regulates the immune system. Consequently, B7-H5 and other immunomodulatory receptors are not the same [40]. B7-H5 is used as both a ligand and a receptor, having an identical inhibitory function [41]. When used either by itself or in conjunction with another immune checkpoint inhibitor, B7-H5 represents a potential novel target for cancer immunotherapy either by itself or in conjunction with another immune checkpoint inhibitor [38]. A recent study reported that the most effective cancer treatment could involve combining anti-B7-H5 and anti-PD-1 antibodies, as demonstrated by their effectiveness in a mouse tumor model [42].

Among the 10 B7 family members, we selected three critical B7 family members for further investigation in BrCa, including B7-2, B7-H3, and B7-H5. We constructed a TMA of 63 female BrCa patients and 20 paracarcinoma samples for mIHC analysis to investigate the relationship between the expression of B7-2, B7-H3, B7-H5, and infiltration of CD8^+^ immune cells. We found that B7-H5 displayed a positive correlation with CD8^+^ immune cell infiltrating in BrCa, providing a novel method for distinguishing between “immuno-hot” and “immuno-cold” subtypes in BrCa.

The major findings of this study could offer a strong scientific foundation for clinical diagnosis and therapy assessment. However, the study had several limitations. Initially, an internal cohort was not used to confirm the expression, correlation, and mutation of the B7 family in BrCa. Instead, the same findings were investigated using a public TCGA cohort. In addition, scRNA-seq data cannot discriminate between different cell types, and certain variations could be present in the expression at the mRNA and protein levels. Furthermore, although we suggested a unique BrCa subtype method, it has not yet been utilized in a clinical immunotherapy cohort.

## Conclusion

In summary, we performed a thorough investigation of B7 family members and clinical features, OS, functional enrichments, genomic alterations, and TIME in BrCa. B7-H5 was found to be strongly associated with CD8^+^ immune cell infiltration in BrCa. Consequently, we provided an original strategy to distinguish between the “immuno-hot” and “immune-cold” phenotypes of BrCa: individuals exhibiting high levels of B7-H5 expression displayed an immuno-hot phenotype, which could benefit them from immunotherapy. Altogether, B7-H5 could function as a switch reversing BrCa from “immuno-cold” into “immuno-hot” status.

## Data Availability

No datasets were generated or analysed during the current study.

## Abbreviations

BrCa: Breast cancer
ScRNA-seq: Single-cell RNA sequencing
TCGA: The Cancer Genome Atlas
TIME: Tumor immune microenvironment
TMA: Tumor microarray
MIHC: Multiple immunohistochemical
ICIs: Immune checkpoint inhibitors
NAT: Neoadjuvant treatment
TILs: Tumor-infiltrating lymphocytes
MDSCs: Myeloidal suppressor cells
OS: Overall survival
DFS: Disease-free survival
BP: Biological process
CC: Cellular component
MF: Molecular function
H -score: I Histochemical score
AML: acute monocytic leukemia
CASP-3: Caspase-3
CTLA-4: Cytotoxic T lymphocyte antigen 4
APC: Antigen presenting cells
B -CLL: C B-chronic lymphocytic leukemia
MDS: Myelodysplastic syndrome
